# Inclusion as Assimilation, Integration, or Co-optation? A Post-Structural Analysis of Inclusion as Produced Through Mental Health Research on Peer Support

**DOI:** 10.1177/10497323231163735

**Published:** 2023-03-20

**Authors:** Aimee Sinclair, Sue Gillieatt, Christina Fernandes, Lyn Mahboub

**Affiliations:** 1 School of Allied Health, 1649 Curtin University, Perth, WA, Australia

**Keywords:** inclusion, peer support, Bacchi, mental health

## Abstract

In the last 20 years, research on the inclusion of peer support within mental health settings has burgeoned, paralleling the broad adoption of service user inclusion within policy as a moral imperative and universally beneficial. Despite the seemingly progressive impetus behind inclusion, increasingly peer support workers talk of exhaustion working within mental health systems, the slow rate of change to oppressive values and practices, and ongoing experiences of workplace exclusion. Such experiences suggest differences in the way in which inclusion is produced across different stakeholder groups and contexts. In this article, we adopt Bacchi’s ‘what’s the problem represented to be?’ approach to identify how mental health research, often understood as an a-political activity, produces versions of inclusion. We argue current research predominantly produces inclusion as ‘assimilation’ and ‘integration’. We use critical inclusion, mental health, and survivor scholarship to evaluate the effects these productions have for peer support and peer support workers, finding that both problematise peer support workers and those seeking support. We consider possibilities for more liberatory productions of inclusion, building on the notion of inclusion as ‘co-optation’. Our analysis points to the need for researchers to engage with an uncomfortable reflexivity to enable more emancipatory possibilities regarding inclusion and peer support.

## Introduction

In this article, we consider the role of mental health research in producing problems of ‘inclusion’ relating to peer support (PS), evaluating whether such problematisations ‘replicate or transform the status quo’ for individuals deemed ‘mentally ill’ or ‘mad’ (Russo, 2022, p. 1, see also LeFrancois & Voronka, 2022). Individuals accessing mental health services are often denied control, safety, and dignity. Much of this exclusion has been linked to psychiatrisation; that ‘mental illness’ or ‘madness’ are traits innate to certain individuals, requiring medical intervention and marking them as essentially inferior to others (Burstow et al., 2014; LeFrançois et al., 2013; Russo & Sweeney, 2016). Through psychiatrisation, support becomes ‘treatment’, often imposed involuntarily. Psychiatrisation is linked to other systems of oppression including white supremacy and colonialism, heteronormativity, capitalism, ableism, and patriarchy (Gorman et al., 2013; Joseph, 2019; Piepzna-Samarasinha, 2018; Redikopp, 2021; Ussher, 2011). Such systems contribute not only to experiences of distress but also to who is deemed ‘mentally ill’, effects of such diagnoses, as well as access to, and experiences of ‘treatment’.

In response, the consumer/survivor/ex-patient (C/S/X) movement has long highlighted the right to self-determination and the value of experiential knowledge in conceptualising, and responding to, ‘mental illness’. We use ‘distress’ and ‘madness’ (as a reclaimed term) rather than ‘mental illness’, unsettling the framing of these experiences as purely biological processes, and, particularly for the latter, to highlight the politicised nature of labels. C/S/X advocacy has contributed to de-institutionalisation, moves towards ‘recovery-orientated’ approaches, and involvement of some mental health service users in service design, delivery, and evaluation (Gooding, 2016; Voronka, 2017). These reforms parallel broader policy engagements with social inclusion and rights of individuals to participate in health-care planning and implementation (Spandler, 2007; Wright & Stickley, 2013).

Increasingly, these inclusionary measures involve employment of individuals with lived experience of distress and/or navigating mental health systems, to deliver PS (Voronka, 2017). Practices of PS originated as a response, particularly within Black, Indigenous, brown, and Queer communities, to a lack of safe and humane supports for individuals experiencing distress (Piepzna-Samarasinha, 2018). Unlike support underpinned by psychiatrisation (psy-care), PS challenges dualisms of care provider/receiver, emphasising solidarity, mutuality, and self-determination (Stratford et al., 2019). Whilst historically sitting outside of, and often resisting, mainstream mental health practices, PS is increasingly drawn into such assemblages through ‘peer support work’.

Drawing on wider discourses around social inclusion, employment of peer support workers (PSWkrs) is predominantly understood as a ‘moral imperative’ (Spandler, 2007, p. 3), politically neutral and thus unquestionable (Barlott et al., 2020). Yet, despite this progressive impetus, PSWkrs report workplace exhaustion, slow rates of change to values and practices, and ongoing experiences of exclusion (Byrne et al., 2019; Edan et al., 2021; Irwin, 2017). Such experiences suggest variations to ‘inclusion’ and the effects of such. Thus, whilst the near universal recognition of the importance of including PS within mental health systems marks a significant move towards social justice, closer scrutiny is required regarding ‘inclusion’. We argue, not that ‘inclusion’ is implemented incorrectly but rather multiple versions of ‘inclusion’ are produced through socio-material practices, including research, with varying effects on PSWkrs and mental health practices.

The contestable nature of ‘inclusion’ has been highlighted in other fields including international development (Calkin, 2015; Cooke & Kothari, 2001a; Koehler et al., 2020), critical race/ethnic studies (Peterson & Åkerström, 2014), health and disability (Marshall, 2012; Pereira & Whiteford, 2013; Taket et al., 2009), mental health (Barlott et al., 2020; Davey & Gordon, 2017; Spandler, 2007; Wright & Stickley, 2013), and mental health service user involvement broadly (Voronka, 2016a; Voronka & Costa, 2019). Challenging ‘inclusion’ as a fixed and universal concept, these works highlight ‘inclusion’ as multiple, with versions ‘brought into being through different social and material practices’ (McWade, 2016, p. 62). For example, when social justice movements use ‘inclusion’ to highlight exclusionary systems and practices, as such concerns become popularised, ‘inclusion’ becomes about behaviour change in excluded individuals rather than changing exclusionary relations. Similarly, concepts such as ‘recovery’ and ‘storytelling’ are shaped a-new as they become entangled with mainstream forces (Barlott et al., 2020; Costa et al., 2012; Harper & Speed, 2012; McWade, 2016; Morrow, 2013).

Whilst objects such as ‘inclusion’ are produced through multiple, entangled practices, for this article, we focus on ‘inclusion’ as produced through mental health research on PS. This body of research has burgeoned in the last 20 years, with literature reviews examining ‘barriers to implementation’ (Vandewalle et al., 2016, p. 234), ‘influences on implementation’ (Ibrahim et al., 2019, p. 285), and ‘facilitators of peer support’ (Kuek et al., 2021, p. 1). Challenging assumptions of research as a neutral, ‘problem-solving’ process investigating pre-existing problematic situations, we consider research as social production (Bacchi, 2012; Mol, 2002; Oliver, 1992), actively shaping ‘the reality we study…with political consequences’ (Rönnblom, 2012, p. 123). We challenge ‘inclusion’ as a fixed ‘problem’, existing independent of research. Rather, research (re)produces specific knowledge, assumptions and ‘problems’ of ‘inclusion’ and ‘peer support work’, and proposes ‘how we ought to proceed’ from such problems (Bacchi & Goodwin, 2016, p. 28). Power operates through such problematisations, governing how one should act, feel, be (Bacchi & Goodwin, 2016). Problems of ‘inclusion’ thus have implications for how PSWkrs are understood by others and themselves and are interacted with and, consequently, how workers support those accessing mental health services.

To consider how ‘inclusion’ of PSWkrs is continually produced through research, and the effects of such, we utilise Bacchi’s (2009) ‘what’s the problem represented to be?’ (WPR) approach. Foucauldian in nature, WPR unsettles conceptual logics of problem representations and how they come about. However, unlike a Foucauldian non-normative analysis, WPR aims to evaluate effects of such problematisations; ‘tak[ing] the side of those who are harmed’ (Bacchi, 2009, p. 42). We adopt an explicitly political lens grounded in the C/S/X movement (Beresford & Russo, 2021) and draw on critical mental health and inclusion theorising to focus on the potentially limiting effects of ‘inclusion(s)’. WPR has been applied to a range of governmental and non-governmental technologies, including the DSM-5 (Buller et al., 2022), media representations (Atkinson et al., 2019), interview transcripts (Lancaster et al., 2017), and symposia (Månsson & Ekendahl, 2015). Yet, despite the role of research in knowledge production, and therefore governing, the application of WPR to academic literature is limited. Martin and Aston (2014) are a notable exception, using WPR to critically analyse representations of women in drug-related research. Our analysis, therefore, is also useful for considering the potential of WPR as a tool to examine political effects of research.

As Mad-identified scholars and allies, unsettling inclusion sits uncomfortably, given its status of moral imperative, achieved through significant social justice advocacy. We do not wish to undermine valuable advocacy that has seen inclusion brought to mainstream political agendas, the progressive effects of inclusionary measures, and the work of scholars who are continuously working towards social justice. It is this work that enables our analysis. However, we believe unsettling enactments of ‘inclusion’ is a discomfort worth sitting with to continue moving towards achieving dignified and socially just mental health services.

## Approach

Using WPR, our analytical focus was on unsettling the ‘taken for granted’ nature of ‘inclusion’ (Barlott et al., 2020, p. 1328), such that we might ‘imagine together alternative ways of understanding and being in the world’ (Tseris et al., 2022, p. 722). As such, our analysis is not representational; we did not aim to identify all peer-reviewed literature, nor to assess quality. Rather, we undertook a broad search to identify multiple ways in which the ‘problem’ (of including PS in mental health settings) is constructed through research.

The first author undertook the literature search using three databases (ProQuest, PsycINFO, and Web of Science), searching 2012–April 2022. We adapted terms from Watson’s (2017) search on PS mechanisms. Whilst aiming to capture a broad range of literature, terms are not exhaustive:

Peer support OR peer support workers OR Peer expert OR lived experience OR Lived expertise OR Consumer survivor OR Consumer provider OR Peer specialist OR Peer worker (title)

AND

Inclusion OR Implementation OR employment OR Co-option OR Authenticity OR Integrity OR Commodification (abstract)

AND

Mental health OR mental illness OR distress OR mental disorder OR schizophrenia OR depression OR personality disorder OR OCD OR eating disorder OR anorexia OR bulimia OR addiction OR psychosis (abstract)

Exclusion criteria included informal/unpaid, child/youth, physical health, online, carer/family supports, and articles unavailable in English. Where articles referred to ‘peer work’ (as a broader spectrum of roles utilising lived experience), we used detail in articles to ascertain whether authors were referring to PS. Articles were also identified through hand-searching reference lists and pre-existing networks.

The first author screened articles for relevance and then used a spreadsheet to record theoretical and methodological approaches, stated aims, proposed solutions, and whether authors reported having lived experience. Recommendations (‘solutions’), reported aims (‘problems’), and ways in which PS work/ers were discussed were coded thematically, enabling the ‘problem’ of ‘inclusion’ as represented in the literature to be identified (step one of WPR). The three problematisations identified were then explored in detail by drawing in additional articles identified from the search. Twenty-seven articles were analysed. The following guided our analysis, adapted from Bacchi and Goodwin (2016):

• What assumptions (regarding inclusion) underlie this representation of the problem?
• What research practices, processes, and paradigms contribute to this representation?
• What is left unproblematic in this problem representation?
• What effects are produced for PS through these representations of the ‘problem’?

We used critical mental health and inclusion literature, alongside comparative analysis (bringing problematisations into dialogue with one another), to identify limits of each and support an imagining otherwise (Rönnblom, 2012). Whilst the analysis was primarily conducted by the first author, all authors met regularly to deepen the analysis. Our multiple and differing subject positions, including our positionings as peer workers (first and fourth authors), and social workers (second and third authors) were productive for facilitating ‘uncomfortable reflexivity’ (Pillow, 2003, p. 175).

Whilst we name specific articles and methods as contributing to problematisations, these should not be understood as independent nor fixed. The nature of research assemblages comprising relations between the object of research, research tools, theoretical frameworks, literature, data, researchers, ethics committees, and academic journals means one singular element does not contribute to these problematisations. Rather, it is the continual entanglement between elements that produce potential problemisations (Fox & Alldred, 2015). For example, whilst we name positivist approaches as contributing to the limits of one problematisation, such approaches are not inherently problematic. Positivist tools may be utilised in struggles for social justice (Gillborn et al., 2018). Rather, it is the intra-action of forces within this assemblage that produce such effects. Similarly, articles often reflected and produced multiple problematisations; the groupings we use in our findings are of problematisations, not articles. Our aim, in highlighting the multiplicity and ever-shifting nature of ‘inclusion’, was to engage with, and push past limits, rather than critique specific articles or ‘fix’ inclusion into three immutable versions produced by specific research elements.

Ethics approval was provided by Curtin University ethics office (HRE2019-0152) as part of a larger project exploring the politics of PS inclusion within mental health systems (Sinclair et al, in press; Sinclair et al., 2023).

## Producing Inclusion

We identified three problematisations of ‘inclusion’ within the literature: ‘inclusion’ as ‘assimilation’, ‘integration’, and ‘co-optation’. We outline each below, bringing them into dialogue with one another, before considering how research might move further towards ‘transform[ing] the status quo’ (Russo, 2022, p. 1).

### Inclusion as Assimilation

This first problematisation constructs the ‘problem’ of ‘inclusion’ as a lack of evidence for PS ‘efficacy and effectiveness’ (Mahlke et al., 2014, p. 276). The increasing employment of PSWkrs is constructed as an effect of C/S/X advocacy and political manoeuvring, with ‘evidence demonstrating effectiveness… lagg[ing] behind’ (Bellamy et al., 2017, p. 161). That is, PS ‘inclusion’ is constructed as a political issue occurring despite quality ‘a-political’ research. The solution thus lies in ‘high quality trials’ (Lyons et al., 2021, p. 315), demonstrating whether PS ‘treatment’ (Castellanos et al., 2018, p. 1) or ‘interventions’ (Bellamy et al., 2017, p. 162; Lyons et al., 2021, p. 315) resolve ‘psychiatric symptoms’ (Fan et al., 2018, p. 2) or improve ‘patient’ self-management (Simpson et al., 2014, p. 14), and thus should be ‘included’ (e.g. Bellamy et al., 2017; Chinman et al., 2014) or not (e.g. Lloyd-Evans et al., 2014).

Given the dominance of the positivist paradigm in medical research (Bacchi, 2016; Beresford & Rose, 2009) and entanglement of distress with medical discourses (Russo, 2022; Speed, 2006), positivist approaches were unsurprisingly dominant in producing this problematisation. A positivist paradigm asks ‘what works?’ (Bacchi, 2016; Gillard, 2019), gathering ‘evidence’ about a singular reality. Such entanglements produce the problem of ‘mental illness’ as internal to the ‘patient’. Thus, PS becomes ‘treatment’. Clinical measures within randomised controlled trials and meta-analyses become the gold standard for measuring peer effectiveness. Whilst some measures, such as goal attainment (Fan et al., 2018), ‘self-management of difficulties’, ‘social functioning, and ‘improved relations with providers’ (Chinman et al., 2014; Lloyd-Evans et al., 2014; Pitt et al., 2013; Smith et al., 2017; White et al., 2020), suggest a widening of outcomes, these were produced as ‘distance travelled’ towards clinical recovery, rather than ends in themselves. Many, such as cost efficacy (Castellanos et al., 2018; Simpson et al., 2014), are clearly defined by needs of government or service providers, not service users. Such outcomes decontextualise understandings of distress and support, silencing, for example, social factors that influence outcomes (King & Simmons, 2018).

This problematisation produces ‘inclusion’ as ‘assimilation’ (Renzaho, 2009, p. 118). PSWkrs, as a condition of ‘inclusion’, must enact ways of thinking and working that align with psychiatric authority and produce clinical outcomes. As argued by Adams (2020) and Gillard (2019), PSWkrs become ‘mini-clinicians’, educating about illness management to intervene and treat individual pathology through psychological education, case management, and risk assessments. PS becomes about ‘individual identity and experience markers: diagnosis [and] history of accessing services’ in supporting psy-outcomes (Voronka & Costa, 2019, p. 7). Notably, these trials and meta-analyses often report PS is ‘unlikely to improve clinical outcomes’ or, at best, is no worse than clinicians (Lloyd-Evans et al., 2014; Pitt et al., 2013). Alternative practices that PSWkrs contribute, specifically those challenging psychiatrisation, are excluded, whilst dominant mental health practices, underpinned by biomedical logic and notions of clinical recovery, are left unquestioned. Thus, ‘inclusion’, through this problematisation, involves PSWkrs having limited control, reflecting what has been variously described in other fields as a ‘top down’ (Cooke & Kothari, 2001a, p. 5) or ‘consumerist approach’ (Beresford, 2002, p. 96). More worryingly, PSWkrs are potentially implicated in oppressive practices associated with ‘treatment’. Oppression not only continues unchallenged but also becomes validated via ‘inclusion’ as ‘assimilation’.

### Inclusion as Integration

The second problematisation involves *how* to include PSWkrs into mental health systems. Unlike the previous problematisation questioning the value of ‘inclusion’, here ‘inclusion’ is constructed as a foregone conclusion, self-evidently desirable and unquestionable. PS practices are produced as unique and valuable (to varying degrees), alongside mainstream services. Challenging the assimilationist version of inclusion, maintaining the ‘authenticity’ of PS becomes important (Rebeiro Gruhl et al., 2016, p. 78). The problem instead is constructed as a technical issue of overcoming ‘barriers’ to ‘inclusion’, whereby such barriers are produced as individual traits, attitudes, and practices.

Elsewhere, this problematisation has been attributed to forces including the rise in popularity of value-based recovery models and critiques of biomedical dominance (Barlott et al., 2020), third wave politics (Béland, 2007; Davies, 2005), and the social model of disability (Marshall, 2012). We add to this critical realist and interpretive research approaches, predominant within literature producing this problematisation. This problematisation was produced predominantly within articles exploring experiences of ‘inclusion’, acknowledging multiple interpretations, including PSWkr interpretations, of a single ‘problematic’ situation (Bacchi, 2016).

Whilst recognising PS voices, by producing ‘inclusion’ as universally beneficial, there is largely only space for beneficial effects of ‘inclusion’. Benefits to PSWkrs were conceptualised as evidence of effectiveness, important because of their contribution to the worker’s ‘recovery’. Ahmed et al. (2015), for example, position ‘fostering the vocational advancement’ of PSWkrs as important, not because this work has important social justice implications but because it could ‘enhance their experiential recovery and community functioning’ (p. 424). Thus, PSWkrs, alongside those they support, are positioned as requiring ‘fixing’, with PS work an effective way of doing so.

By providing ‘evidence’ of PS effectiveness, the critical realist paradigm contributes to the first problematisation identified. However, whereas the value of ‘inclusion’ was questioned in the former, the critical realist approach was more central to producing ‘inclusion’ as a foregone conclusion, universally beneficial. Negative experiences of ‘inclusion’ reported by PSWkrs, such as emotional harm to PSWkrs or resistance from non-peer disciplines, were often glossed over, or problematised as technical issues. That is, through the production of negative experiences as technical issues needing resolution, the universal benefit of ‘inclusion’ remains intact.

These ‘barriers’ are constructed as two kinds of ‘problems’: ‘problems’ relating to PSWkrs and/or PS work, and ‘problems’ seemingly external to PS. Firstly, PSWkrs are problematised as lacking the disposition to do ‘mental health work’, where non-peer work is upheld as the norm. ‘Problems’ include a lack of professional boundary setting (Mancini, 2018; Rebeiro Gruhl et al., 2016; Vandewalle et al., 2016), ‘residual cognitive, social or emotional limitations’ (Vandewalle et al., 2016, p. 243), ‘relapse’ (Ahmed et al., 2015, p. 430), problems ‘maintaining personal wellness’ (Ahmed et al., 2015, p. 424), limited self-care (Rebeiro Gruhl et al., 2016), and internalised ‘pressure to succeed’ (Otte et al., 2020). Such problematisations produce PSWkrs as ‘other’, needing special accommodations such as lighter workloads, time off, and clinical support (Ahmed et al., 2015), because of ‘mental health needs’ (Mancini, 2018, p. 135). Silenced are the structural issues that emotionally impact all mental health workers, but, in particular, the significant discrimination and moral distress peer workers must navigate due to ‘inclusion’ (Byrne et al., 2019; Edan et al., 2021; Sinclair, 2018). For example, when PSWkr values and ethics are violated through requests to assist with seclusion, restraint, involuntary treatment, and mandatory reporting (Alvarez-Vasquez et al., 2020; Irwin, 2017); non-peer workers, and the system at large, are positioned as benevolent by ‘accommodate[ing]’ peers ‘who became symptomatic’ (Mancini, 2018, p. 134) and by providing peer workers with training around self-care and self-management, supporting what is constructed as an inherent lack in peer dispositions.

PSWkrs are also produced as lacking skills for mental health work. For example, Rebeiro Gruhl et al. (2016) attribute PSWkrs burnout to ‘ambiguous personal boundaries’ (p. 83), suggesting training is required to assist in communicating lived experience, setting boundaries, and self-care. Yet, experiences labelled as ‘burnout’ can also be understood as resulting from other experiences described by PSWkrs in the literature: being the sole peer worker, having to practise in ways that conflict with peer values, exclusionary practices of other workers, and a systemic devaluing of lived experience. ‘Inclusion’ in this sense heightens awareness of difference, solidifying ideas of those occupying PS roles as essentially more vulnerable and less skilled than non-peer workers, and leaving oppressive relations uncritiqued.

Peer support, as a practice, is also problematised as a ‘barrier’. Mancini (2018), for example, describes ‘the very things that make peers unique and effective may also contribute to the confusion and apprehension they experience’ (p. 135). Such problems include the ‘emotional stress of helping others’ (Ahmed et al., 2015, p. 434), conflicting identities (Otte et al., 2020; Vandewalle et al., 2016), a lack of ‘role clarity’ or ‘role confusion’ (Kuek et al., 2021; Mancini, 2018; Otte et al., 2020; Rebeiro Gruhl et al., 2016; Vandewalle et al., 2016), and lack of professional standards (Otte et al., 2020; Vandewalle et al., 2016). Here, defining features of PS, such as affective relations involved in ‘being with’ struggle, and co-creating care (Dragojlovic & Broom, 2018; Eales & Peers, 2021; Fritsch, 2010) are problematised because they fail to adhere to psy-enactments of care. As above, solutions result in PSWkrs becoming like existing mental health workers through training, standardisation, and credentialling.

Alternatively, problems external to PSWkrs include stigmatisation by, or hostility from, non-peer staff (Ahmed et al., 2015; Kuek et al., 2021; Mancini, 2018; Otte et al., 2020; Rebeiro Gruhl et al., 2016; Vandewalle et al., 2016), unsupportive work environments (Kuek et al., 2021; Mancini, 2018) with ‘untenable productivity standards’ (Ahmed et al., 2015, p. 431), poor compensation and employment opportunities (Ahmed et al., 2015; Vandewalle et al., 2016), and staff not knowing how to ‘accommodate’ peers ‘who became symptomatic’ (Mancini, 2018, p. 134). Whilst some ‘barriers’ reflect a continuing problematisation of PSWkrs, others allude to exclusionary relations within mental health systems. Yet, rather than acknowledge these relations, they are attributed to lack of ‘organizational readiness’ (Mancini, 2018, p. 136) and non-peer worker understandings. Clear policies and procedures, related to ‘paperwork, confidentiality, professional boundaries’ (Mancini, 2018, p. 135), are proposed as solutions to a lack of ‘organisational readiness’, suggesting that PSWkrs need guidelines to abide by dominant practices. The alternative, that such policies and practices may be problematic, is silenced. Similarly, where peers report experiencing microaggressions from non-peer staff (Bailie & Tickle, 2015; Edan et al., 2021; Sinclair, 2018), this is often attributed to staff misunderstandings of the peer role, suggesting that any fear non-peer staff have that psy-enactments may need to change is unfounded. The ‘problems’ are resolved through education and role clarity, such as clearer job descriptions (Mancini, 2018; Otte et al., 2020). In other words, when non-peer staff may feel their jobs are under threat (Gillard et al., 2013), or practices are being critiqued, the solution is to re-assure staff that this is not the PSWkr role. The idea that PSWkrs may have valid critique of psy-care is put to one side. Instead, PSWkrs are positioned as an ‘add-on’ to existing service delivery (Edan et al., 2021).

‘Inclusion’ is thus produced as ‘integration’ (Renzaho, 2009, p. 118). Whilst PS practices are positioned as valuable, only certain aspects of lived experience is again ‘included’, those complementing existing supports, such as an ability to act as a ‘coping model’ and tell ‘recovery stories’ (Ahmed et al., 2015, p. 425), to facilitate goal setting and teach ‘relapse prevention skills’ (Smith et al., 2017, p. 387). As with the first problematisation, the knowledge PSWkrs bring as an ‘alternative epistemology to the medical and psychiatric paradigm’ is excluded (Edan et al., 2021, p. 3274). Mainstream values, knowledges and practices, and the oppressive forces that shape these remain largely unproblematised. The forces involved with who is marked as needing to ‘recover’ or not, and what it means to ‘recover’ are left unquestioned, despite literature highlighting limits to conceptualisations of recovery (Barlott et al., 2020; Fullagar et al., 2019; Harper & Speed, 2012; McWade, 2016; Morrow, 2013).

Such enactments of ‘inclusion’ as ‘integration’ were present regardless of whether it was non-lived experience researchers presenting solutions, or PSWkrs themselves. For example, Mancini (2018) describes how peers identified that they ‘required continuing education in…clinical diagnosis…suicide risk assessment… and resource management (case management)’ (p. 213), reflecting an individual framing of barriers to inclusion, and also a privileging of clinical ways of practicing. This highlights one of the limitations to critical realist research and ‘inclusion’ produced through such research; despite ‘inclusion’ of lived experience voices, by assuming sovereign actors that sit outside of discursive-material relations, there is no consideration of how PSWkr meaning-making is socially constituted, mediated by dominant enactments of inclusion and care.

‘Inclusion’ as ‘integration’, whilst providing possibilities for self-determination when compared to ‘inclusion’ as ‘assimilation’, still has potential to ‘conceal and reinforce oppressions and injustices’ (Cooke & Kothari, 2001b, p. 13) by constructing inclusion as a state of being that can be ‘treated’. In some ways, this enactment is more insidious and harder to disrupt, given its construction as universally beneficial, alignment with ‘recovery’, and thus a ‘moral imperative’ (Spandler, 2007, p. 3; Voronka & Costa, 2019). Any limiting experiences reported by PSWkrs and individuals accessing supports are reduced to technical rather than political problems, creating the illusion that exclusion and dominance are a non-issue (Telleria, 2020).

### Inclusion as Co-optation

This final problematisation, which we have drawn on in exploring limits of previous problematisations, involves a critique of dominant enactments of ‘inclusion’. Through ‘inclusion’, the psy-complex ‘co-opts’ peer bodies, stories, experiences, values (Adams, 2020; Alberta & Ploski, 2014; Stamou, 2014; Stratford et al., 2019; Voronka, 2017), and our desires to ‘make a difference and support people in a different way’ (Irwin, 2017, p. 154) and excludes individuals such as those who cannot meet entry requirements for ‘formalised’ PS (Adams, 2020). Inclusion benefits psychiatry: PS becomes about achieving the aims of psy-care rather than challenging oppressive practices. Dominant enactments of ‘inclusion’ do little to ‘disrupt structural violence, and rather allows psy-powers to proceed’ (Voronka, 2017, p. 336). The system is thus problematised; it co-opts PS’s activist social justice roots as an alternative and challenge to psy-governance. Whereas in other critical mental health discourses, the ‘psy-complex’ is produced as ‘inform[ing] and intersect[ing] with other neoliberal forms of oppression’ (LeFrançois et al., 2016), we found, with some exceptions, that literature producing this problematisation tended to sideline such intersections.

This problematisation is constructed predominantly through research conducted by peer workers, or through collaboration with peer workers throughout the process. Such approaches involve an ‘epistemological and methodological shift away from… [the] paternalistic professional dominance’ (LeFrancois et al., 2016, p. 1) of mental health research. ‘Experiential expertise’ (Adams, 2020, p. 2) of PSWkrs is valued, allowing for alternative understandings and space to consider, for example, the problematic effects of peer employment. Whilst in our experience, these critiques of inclusion are commonly spoken of outside of academia, this problematisation was much less dominant in the literature than former problematisations, which we attribute to the politics of knowledge production, excluding lived experience (Kalathil & Jones, 2016). Research applying critical/Mad theories (IMSC, 2022; LeFrancois & Voronka, 2022) to PS was even less present, as was research attending to perspectives predominantly excluded from PS work (LeFrancois & Voronka, 2022; Spandler & Poursanidou, 2019). Most literature we reviewed, whilst acknowledging the history, importance, and unique values of PS, did not stray far from ‘accepted’ methodological traditions within qualitative (e.g. Adams, 2020) and quantitative research (e.g. Alberta & Ploski, 2014) and did not tend to engage theoretically with issues of power and intersectionality, which contribute to some of the limitations we explore below.

Stemming from essentialist notions regarding ‘inclusion’ and ‘peer support’, this problematisation produces an ‘authentic’ form of PS becoming subsumed through ‘inclusion’. Most of the literature we reviewed documents a gradual shift; away from an authentic version of PS described as ‘user led’, ‘grass roots’ (Stamou, 2014, pp. 167, 173), and ‘true’ (Adams, 2020, p. 4) towards ‘professionally led’, ‘medicalising’ (Stamou, 2014, p. 173), and ‘institutionalised’ (Adams, 2020, p. 1) support. ‘Inclusion’ ‘fundamentally changes the scope and practice of the work itself’ (Adams, 2020, p. 4), which is produced as problematic; it results in loss of integrity (Adams, 2020; Irwin, 2017; Stamou, 2014; Stratford et al., 2019) and devaluing of lived experience expertise and values (Adams, 2020). Such essentialist narratives of co-optation are critiqued across other areas of social justice, including Mad studies more broadly, for producing fixed, monolithic, and often romanticised identities and collective politics existing a-historically (Calkin, 2015; Diamond, 2013; Gorman et al., 2013; LeFrançois et al., 2013; Roy, 2017), thus sidelining both the diversity and dynamic nature of ‘peer support’ and ‘inclusion’.

For example, a failure to engage with issues of race, gender, class, sexuality, or diagnostic differences crosses over all three problematisations, resulting in the continued production of a white, western, predominantly ‘sane-performing’ PSWkr (Diamond, 2013; Gorman et al., 2013; Joseph, 2019; Redikopp, 2021; Voronka, 2016b). Histories of ‘authentic’ PS, often attributed to the C/S/X movement of the 1970s (Adams, 2020; Irwin, 2017; Stratford et al., 2019), silence the long history of mutual aid present within Black, brown, Indigenous, and Queer communities, and expertise challenging the colonial project (Kalathil & Rose, 2019). They also ignore the differential effects of taking up a peer identity and ways in which only certain bodies are ‘recognized as a viable “lived experience” subject’ and thus ‘included’ (Voronka, 2016b, p. 197). To speak of the limitations of the first two problematisations can be considered a position of privilege which necessitates critical reflection and engagement with the experiences and expertise of ‘marginalized and minority groups (who) have not had a substantial role in involvement initiatives’ (Kalathil, 2013, p. 122). Similar arguments can be made for other axes of oppression. For example, universalising the impact of ‘inclusion’ excludes consideration of ways in which gender and race intersect with how ‘care’ is valued within mental health systems. Without critically attending to these intersections, ‘scholars potentially…maintain the systems of psychiatric violence they seek to undo’ (Redikopp, 2021, p. 98).

A co-optation problematisation also has potential to erase dissent existing within the C/S/X movement regarding the ‘problem’, what Spandler and Poursanidou (2019) refer to as ‘mad specific’ exclusions (p. 3). Cresswell and Spandler (2016, see also Daya et al., 2020) highlight that for Mad studies, ‘the psy-regime swallows up both psychiatry and medicine and is the source of all harm; it cannot be reformed’ (p. 369). Yet, for psychopolitics, ‘psychiatry and medicine are part of a hard-fought-for welfare state. They may be criticised and reformed but must be defended’. Within a co-option narrative, PSWkrs become essentialised and binarised: as either radical activists independent of the system, or co-opted workers, ‘seduced by inclusion’. This positioning erases the complexity in which we act and the effect of such acts, ways in which individuals are continually ‘becoming’, shaped by various forces, the various ways individuals ‘included’ have reformed psychiatric systems, and how PS has always existed within politics. For example, arguably, the C/S/X movement ‘co-opted’ historical practices of mutual aid from Black communities, yet this is not considered co-optation but rather as contributing to the emergence of formal ‘peer support work’. Similarly, histories of PS as emerging from the employment of people who had been committed in Parisian asylums in the late 18th century (see, for example, Stratford et al., 2019) suggest a history of PS being practised from within, and as part of, formal mental health systems, contradicting an ‘authentic’ PS existing a-historically beyond politics and dominant systems. Further, psychiatry and other mental health fields are not homogenous or monolithic (Rose, 2017), nor are the workers within such fields, yet there is a tendency to subscribe to these essentialist notions through ‘co-optation’.

## Implications for Future Research

Our analysis highlights two dominant ‘problems’ of ‘inclusion’ produced *through* research, as well as a third, less prevalent, problematisation. We have argued that despite potentially liberatory intentions, ‘inclusion’ as constructed within mental health research positions PSWkrs and PS work as the problem, producing and reinforcing forces of psychiatrisation alongside colonialism, patriarchy, and other systems of oppression. Our analysis contributes to a growing body of work highlighting the perpetration of epistemic injustice for individuals deemed ‘mentally ill’ by mainstream mental health research (LeBlanc & Kinsella, 2016; LeFrancois & Voronka, 2022; Russo, 2022). PSWkrs are afforded little power through the subjective effects of dominant notions of ‘inclusion’; our experiences and identity reduced to ‘fixing’ ourselves and others. Categories of ‘us’/‘them’ (‘sane’/‘mad’) are minimally disrupted. Rather, some, who are able to, or willing to, perform as ‘viable “lived experience” subject(s)’ (Voronka, 2016b, p. 197) are enabled to step inside the circle of ‘inclusion’, whilst others remain excluded. Peer support becomes something a-new, potentially replicating psy-enactments of care. Yet, those who are ‘included’ risk being excluded from critical/radical survivor communities through constructions of ‘inclusion’ as ‘co-optation’.

While beyond the article’s scope, we suggest multiple ‘inclusions’ are similarly enacted in services, contributing to oppressive tensions experienced by PSWkrs, including experiences of being constantly monitored, over-managed, exclusion, and moral distress (Edan et al., 2021; Irwin, 2017). PSWkrs are pulled between wanting to practice in anti-oppressive ways and critiquing oppressive practices whilst needing to exist within a system that so readily excludes, and thus needing to be grateful for ‘inclusion’. ‘Inclusion’ has potential to replicate and transform the status quo in complex ways for PSWkrs and individuals accessing services, ways that currently go largely unexamined.

As part of the research assemblage producing these problematisations, it is critical researchers engage in ‘self-problematisation’ (Bacchi & Goodwin, 2016, p. 20), considering how our own acts are productive of ‘inclusion’ and the effects of such. Self-problematisation involves an ‘ethics of discomfort’, subjecting one’s own positionality to critical attention to highlight ‘the extent to which we may inadvertently be complicit in oppressive modes of governing’ (Cooke & Kothari, 2001b, p. 15). Such discomfort is vital in ensuring we move beyond the narrowness of epistemological awareness, to a ‘genuine and rigorous reflexivity’ (Cooke & Kothari, 2001b, p. 15).

In working to examine limitations of the third problematisation, where we position our own analysis and attempt to stand in solidarity with lived experience, we have aimed to engage in such self-problematisation. We do so recognising that, as researchers, we are just one element co-produced within a research assemblage favouring particular ways of thinking, feeling, and doing research. However, this should not absolve us of ‘our responsibility for what we create within the radius of our own projects, no matter how limited that radius might appear’ (Russo, 2022, p. 3).

How then, might we progress past some of the limitations within current productions of ‘inclusion’? How can we recognise complexity, difference, and diversity in new and different ways that move us beyond fixed notions of ‘inclusion’? Russo (2022) suggests we might accept forces of psychiatrisation and co-optation as unavoidable as we progress towards emancipation. Using Foucault’s notion of the apparatus, as suggested by Swerdfager (2016), post-humanist theorising such as Deleuze’s concept of ‘assemblages’ (Barlott et al., 2020) or intersectional/postcolonial theorising (Roy, 2017) may provide potential to do so, enabling a more nuanced understanding of the multiplicity of socio-material practices passing as ‘inclusion’, and how these produce certain enactments of PS, always in motion, always ‘becoming’. This involves understanding co-optation not simply as an action but ‘rather an emergent process in which there is always some change, and for some of us, there emerge ethical/political concerns with this change’ (Roy, 2017, p. 256).

Moving away from notions of ‘authentic’ PS, we might instead explore how various ‘inclusionary’ forces impact on PS in different ways in different contexts, and what effect these have across intersections of gender, class, diagnosis, and other axes of oppression. Rather than arguing whether PSWkrs should be engaging with mental health systems from the ‘inside’ or ‘outside’, we might consider the productive potentials and limitations within both; considering where and when ‘exclusion’ allows freedom of thought and an ability to challenge the status quo (Labonte, 2004; Mahboub, 2019), as well as when, where, and to whom certain enactments of ‘inclusion’ may be useful. In this article, we have considered the role of research in such enactments, yet research is just one part of a larger assemblage of bodies, physical environments, temporalities, affects, policies, and practices producing ‘inclusion’ as multiple.

## Conclusion

By highlighting the diverse ways in which ‘inclusion’ is produced through research, we have aimed to unsettle not only enactments of ‘inclusion’ that go predominantly uncontested but also research as a ‘neutral, “problem- solving” and largely technical process’ (Marshall, 2012, p. 55). We have argued research actively intervenes in the production of ‘inclusion’ and ‘peer support’ work. We have highlighted how dominant notions of ‘inclusion’ produced through research have potentially delimiting effects for PSWkrs and leave oppressive power relations unexamined. An alternative enactment of ‘inclusion’, constructed predominantly through Mad/survivor research, provides potential to move us closer to social justice outcomes if we continue to engage with its limitations and do not allow it to solidify. This critical reflection on the role of research in the production of ‘inclusion’, whilst often uncomfortable, is integral for ensuring, as researchers, we are aware of the problematic effects of our own well-intentioned efforts, and thus how we might continually adjust our practices such that we move closer to achieving justice and equality for individuals with lived experience of distress and/or psychiatric oppression.
